# Are Antimicrobial Peptide Dendrimers an Escape from ESKAPE?

**DOI:** 10.1089/wound.2019.1113

**Published:** 2020-06-09

**Authors:** Yayoi Kawano, Olivier Jordan, Takehisa Hanawa, Gerrit Borchard, Viorica Patrulea

**Affiliations:** ^1^ Laboratory of Preformulation Study, Faculty of Pharmaceutical Sciences, Tokyo University of Science, Chiba, Japan.; ^2^ Institute of Pharmaceutical Sciences of Western Switzerland, University of Geneva, Geneva, Switzerland.

**Keywords:** chronic wounds, ESKAPE microbial infection, topical antimicrobials, chitosan derivatives, antimicrobial peptide dendrimers, nanoparticles

## Abstract

**Significance:** The crisis of antimicrobial resistance (AMR) increases dramatically despite all efforts to use available antibiotics or last resort antimicrobial agents. The spread of the AMR, declared as one of the most important health-related issues, warrants the development of new antimicrobial strategies.

**Recent Advances:** Antimicrobial peptides (AMPs) and AMP dendrimers (AMPDs), as well as polymer dendrimers are relatively new and promising strategies with the potential to overcome drug resistance issues arising in ESKAPE pathogens (*Enterococcus faecium, Staphylococcus aureus, Klebsiella pneumoniae*, *Acinetobacter baumannii*, *Pseudomonas aeruginosa*, and *Enterobacter* species) colonizing chronic wounds.

**Critical Issues:** AMPs–AMPDs suffer from limited efficacy, short-lasting bioactivity, and concerns of toxicity. To circumvent these drawbacks, their covalent coupling to biopolymers and/or encapsulation into different drug carrier systems is investigated, with a special focus on topical applications.

**Future Directions:** Scientists and the pharmaceutical industry should focus on this challenging subject to either improve the activity of existing antimicrobial agents or find new drug candidates. The focus should be put on the discovery of new drugs or the combination of existing drugs for a better synergy, taking into account all kinds of wounds and existing pathogens, and more specifically on the development of next-generation antimicrobial peptides, encompassing the delivery carrier toward improved pharmacokinetics and efficacy.

**Figure:**
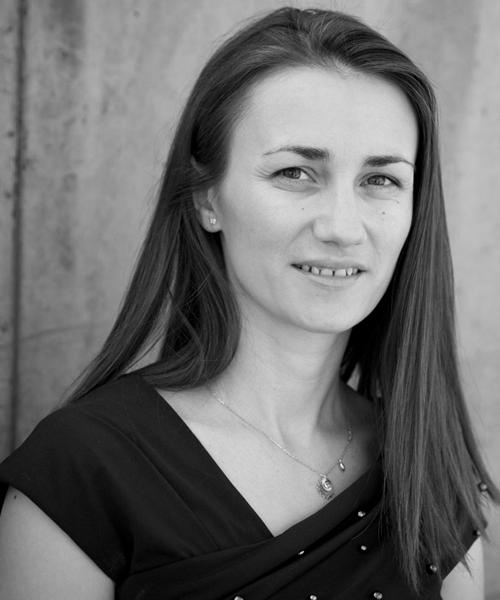
**Viorica Patrulea, PhD**

## Scope and Significance

Microbial infection has become a major global threat due to the emergence of antimicrobial resistance (AMR). This spurred the need for innovative strategies to fight multidrug-resistant (MDR) bacteria.

This review summarizes the most relevant available antimicrobial agents related to topical therapy. We discuss antiseptics and antibiotics commonly used in wound care and summarize the shortcomings of their application in certain patients. Furthermore, we review innovative strategies relying on linear and dendrimeric antimicrobial peptides (AMPs), including the drug delivery approaches that may improve their efficacy against AMR.

## Translational Relevance

Treatment of infections resulting from bacteria belonging to the ESKAPE (*Enterococcus faecium, Staphylococcus aureus, Klebsiella pneumoniae*, *Acinetobacter baumannii*, *Pseudomonas aeruginosa*, and *Enterobacter* species) collection is a clinical challenge. This article describes commercially available topical antimicrobials and antiseptics, emphasizing their bacterial activity and limitations. Key AMPs tested in clinical trials are presented. To cope with the urgent need for innovative solutions, novel dendrimer peptides, chemical conjugates, and nanocarriers are discussed, which may allow for an improved activity against MDR bacterial infections.

## Clinical Relevance

The care and management of infected wounds is a burden not only for patients, who suffer from severe pain, but for clinicians as well. AMR remains a critical issue and novel AMPs and AMP dendrimers (AMPDs) offer solutions for eradicating MDR bacteria, allowing healing to occur.

## Background

Skin has essential roles in maintaining homeostasis, preventing microbial invasion, and providing a barrier between body tissues and the external environment. However, burns, and traumatic or chronic wounds, such as venous, diabetic, and pressure ulcers, compromise the protective barrier that skin offers and subsequently facilitate the risk of bacterial infections.^1^ Microbial infection due to AMR is one of the major global threats that continues to worsen despite the efforts in finding solutions. Around 0.7 million people die each year due to the emergence and persistence of MDR bacteria or so-called superbugs.^2^ It is estimated that by the end of 2050, AMR could lead to more than 10 million deaths annually, compared with more than 8.2 million deaths attributable to cancer.^2^

MDR hinders the healing process in wounds, as most of the wounds would develop infections at some point.^1^ One of the most challenging MDR bacteria is *P. aeruginosa*, which is part of the problematic bacterial collection called ESKAPE. Among other virulence factors such as adhesion, quorum sensing, or toxin production, the success of ESKAPE pathogens in escaping from the antibiotic treatment lies in the different posttranslational modifications found in their proteome.^3^ With the emergence of multidrug-resistant bacteria and reemerging infectious diseases, development of new antibacterial agents has become an urgent task.

The World Health Organization (WHO) called on member states to act against AMR since 1998. WHO calls for improvement of surveillance and testing systems, appropriate use of antibacterial agents, and strengthening countermeasures against infectious diseases. WHO has recently listed nosocomial ESKAPE among the 12 families of MDR bacteria. Moreover, AMR negatively affects the global economy and especially poor countries where the health care system is underdeveloped.^4^ Recent calculations estimate that by the end of 2050, global MDR costs will go above $100 trillion.^2^

Clinical and Laboratory Standards Institute (CLSI) guidelines established a list of available antibiotics specifically active against ESKAPE bacteria (Table 1). Surprisingly, many antibiotics and combination of antibiotics have been removed from the list due to their side effects or lack of efficacy. Even for relatively new antibiotics, incidences of resistance are reported and for some bacteria, such as *A. baumannii*, *K. pneumoniae*, and *P. aeruginosa*, resistance has not yet been overcome. These latter were generally kept in the CLSI guideline. Some antimicrobial agents were added since 2010 due to evidence of absence of resistance against specific strains.

**Table 1.** Antimicrobial agents against ESKAPE added, removed, or kept from the CLSI guidelines over the period 2010–2019

Pathogen	Available Antibiotics	Efficacy*–*Resistance Reported	Ref.
*Enterococcus faecium*	Dalbavancin, Oritavancin, Tedizolid, Telavancin	No resistance reported	^5^
	Vancomycin	Resistance found	
*Staphylococcus aureus*	Dalbavancin, Oritavancin, Tedizolid, Telavancin	No resistance reported	^6,7^
	Ceftaroline	Resistance found	
	Amikacin, Amoxicillin–clavulanate, Ampicillin–sulbactam, Cefaclor, Cefamandole, Cefazolin, Cefdinir, Cefepime, Cefmetazole, Cefonicid, Cefoperazone, Cefotaxime, Cefotetan, Cefpodoxime, Cefprozil, Ceftazidime, Ceftizoxime, Ceftriaxone, Cefuroxime, Cephalothin, Doripenem, Ertapenem, Kanamycin, Imipenem, Loracarbef, Meropenem, Moxalactam, Netilmicin, Oxacillin, Piperacillin–tazobactam, Ticarcillin–clavulanate, Tobramycin	Removed from CLSI guidelines	
*Klebsiella pneumoniae*	Aztreonam, Cefazolin, Cefepime, Ceftazidime, Cefotaxime, Ceftaroline, Ceftazidime–avibactam, Ceftizoxime, Ceftolozane–tazobactam, Ceftriaxone, Doripenem, Ertapenem, Imipenem, Meropenem	Resistance found	^8^
	Cephalothin, Nalidixic acid, Ticarcillin	Removed from CLSI guidelines	
*Acinetobacter baumannii*	Doripenem, Ertapenem, Imipenem, Meropenem	Resistance reported	^9–11^
	Mezlocillin, Ticarcillin	Removed from CLSI guidelines	
*Pseudomonas aeruginosa*	Colistin, Doripenem, Imipenem, Meropenem, Piperacillin, Piperacillin–tazobactam, Ticarcillin–clavulanate	Resistance reported	^12,13^
	Cefoperazone, Cefotaxime, Ceftizoxime, Ceftriaxone, Moxalactam, Ticarcillin	Removed from CLSI guidelines	
*Enterobacter* spp.	Aztreonam	No resistance reported	^14,15^
	Cefazolin, Cefepime, Cefotaxime, Ceftaroline, Ceftazidime, Ceftazidime–avibactam, Ceftizoxime, Ceftolozane–tazobactam, Ceftriaxone, Doripenem, Ertapenem, Imipenem, Meropenem	Resistance reported	
	Cephalothin, Nalidixic acid, Ticarcillin	Removed from CLSI guidelines	

CLSI, Clinical and Laboratory Standards Institute.

Lastly, AMPs have attracted high interest, since they are less likely to induce MDR. AMPs are short peptides consisting of 10–50 residues and produced by all living forms, including protozoa, bacteria, animals, and humans. They are important mediators of innate immune defense. Their amino acid sequence and secondary structure are diverse, but most of the antibacterial peptides are amphipathic with both basic and hydrophobic clusters, and bacterial cell membranes rich in acidic lipids are used for ATP production. Most AMPs possess cationic properties. They have shown a broad activity against a wide range of pathogens, including MDR bacteria by lysing cell membrane through electrostatic interactions.

However, AMPs are rapidly degraded once in contact with human serum (short plasma half-life), losing their activity and most of them are toxic, besides having high production costs.^16^ Another promising approach is the use of AMPDs, which showed better activity than conventional AMPs, but still show quite high toxicity and poor stability in human serum of only a few hours.^17^ There is therefore an urgent need to develop new strategies of application of these AMPs and AMPDs avoiding their degradation, while reducing their toxicity at therapeutic concentrations. Chemical conjugation of these potent molecules to different polymers may offer a solution to overcome these drawbacks. Still, the exact antimicrobial mechanism of the polymer–peptide conjugate needs to be further evaluated, as there is a lack of clinical studies describing the healing of infected wounds upon application of AMPs or AMPDs.

## Discussion

### Infection and biofilm formation

In general, microorganisms colonize all open wounds, although not all wounds will show clinical signs of infection.^18,19^ Upon infection, microbes create a cytotoxic environment, which often leads to chronic wounds and eventually to gangrene with successive amputation of the infected limb or even to the death of the patient due to sepsis.^20^ However, the likelihood that a wound will be infected is not only related to the presence of the microorganisms, but to the depth, size, and location of the wound, as well. For example, purulent secretions or local expressions of inflammation are clear indications that an infection has occured.^18^ Nevertheless, the inflammation may be caused by conditions unrelated to a wound, such as diabetic neuropathy, venous insufficiency, or ischemia.^20^

As a rule, wounds can be classified as acute or chronic. Acute wounds are a result of an injury, surgery, or the use of intravascular devices. Acute wounds heal within a very short period of time, following successive phases of inflammation, proliferation, migration of keratinocytes and fibroblasts, and final tissue maturation.^21^ Wounds that fail healing within 3 months through the normal healing process are categorized as chronic.^22^ This type of wounds show a persistent inflammation phase, which is characterized by a continuous influx of polymorphonuclear neutrophils leading to impaired wound healing.^23^

Moreover, as wounds grow deeper and become more complex, they can infect the underlying tissue and bone causing osteomyelitis.^24^ For instance, surgical-site infections, such as superficial incisional, deep incisional, organ, or interorgan space infections, will show postoperative signs of infection typically within the first 10 days, occasionally only after month(s).^25^

Diabetic wounds, such as foot ulcer or venous ulcer, result from uncontrolled glycemia, leading to microvascular complications (retinopathy, nephropathy, and neuropathy) and very high incidence of infection.^20,26^ The potential of the wounds to be infected or to heal depends on the surrounding skin and mucous membranes. Actually, the longer the wounds are exposed to the bacteria, the easier it is for the bacteria to proliferate and colonize.

Wounds are ideal hosts for bacterial colonization, providing a warm environment and nutrients. This may lead to local or dangerous systemic infections.^27^ The propensity for a wound to become infected is directly proportional to the pathogenicity or virulence of the microorganism, and inversely related to both local and systemic resistance of the host.^28^ Local factors refer to wound size and depth, degree of chronicity, contamination, type of wound, presence of necrotic tissue, anatomic location, and compromised sterilization of the materials; while systemic factors relate to diabetes, obesity, smoking, age, alcoholism, malnutrition, radiation, medication (with steroids, chemotherapy), or inherited neutrophil defects. Usually, infected wounds are accompanied by foul odor, necrotic tissue, wound pain, and impaired healing. It is generally considered that wound colonization occurs at bacterial loads <10^5^ bacteria per gram tissue and infection when >10^5^ bacteria per gram tissue are found.^23^

Interestingly, critically ill patients have higher rates of MDR microorganisms compared with other patients.^29^ Most common MDR bacteria are methicillin-resistant *S. aureus* (MRSA), vancomycin-resistant enterococci, and MDR Gram-negative bacteria (*i.e.,* extended-spectrum β-lactamases; AmpC-type β-lactamases, and metallo-β-lactamase).^30,31^ Importantly, a rational administration of topical antimicrobial agents should be considered to prevent any resistance development. For this, the use of systemic antibiotics is indicated only when infection is evident or in case of ascending limb infection, sepsis, or incision wounds spreading cellulitis.^32^ Bite wounds, depending on their severity, should be treated with oral antibiotics.^33^ Care should be taken to limit the duration of antibiotic administration than to the recommended one, to avoid the development of AMR.^34^

Bacteria within biofilms are 100–1,000 times more tolerant to antibiotics, disinfectants, or mechanical stresses; thus impeding conventional antibiotic therapy and delaying wound healing in chronic infections.^35^ Pathogenic bacteria amplify the AMR issue by creating a 3D bacterial biofilm network, which can strongly enhance the chronicity of the wounds. Biofilms are formed of communities with a high bacterial cell density that are enclosed in a self-produced matrix of extracellular polymeric substance. This matrix composed of exopolysaccharides, proteins, and DNA confers additional resistance to bacteria.

**Figure 1.**
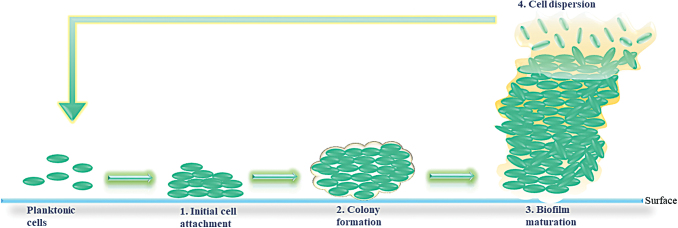
Biofilm formation, including the four stages: (1) Cell attachment, (2) colony formation, (3) mature biofilm, and (4) dispersion of the cells. Color images are available online.

The formation of biofilm (Fig. 1) begins when planktonic (free swimming) cells find their way to a surface to which they attach, followed by their rearrangement to form and maturate the biofilm. Biofilm dispersion, also referred as cell detachment, is followed by release of planktonic bacteria that will restart formation of a new biofilm at distant sites.

Examples of biofilm-related infections include colonization of almost any surface, including abiotic surfaces (hospital walls, medical devices, implants, catheters, etc.), as well as of biotic surfaces (surgical sites, wounds, lungs, urinary tract, cardiac tissues, bones, etc.).^36^

Several mechanisms have been proposed to understand the tolerance of bacteria in biofilms to antibiotic treatment^37^:

1. Limited antibiotic penetration into the biofilm
2. Gene mutation
3. Reduced metabolic rate, growth rate, and division rate
4. Presence of slowly growing persister cells that could reactivate after the antibiotic treatment
5. Overexpression of bacterial efflux pump in biofilm, which leads to increased resistance to antibiotics
6. Protection by the self-secreted matrix of extracellular polymer substance.

There are few antimicrobial agents in clinical use to specifically target biofilms, probably due to the poor understanding of biofilm formation. Resistance ability can be explained if combining the before-mentioned mechanisms. For instance, *P. aeruginosa*, which frequently leads to biofilm-associated infections, adapts easily to the hostile habitat by producing adapted phenotypes and mutations. On the other hand, the geometry of *P. aeruginosa'*s colonies in a shape of tall ridges or wrinkles (referred as colony rugosity), facilitates their oxygen supply and allow them to grow taller.^35^

Current biofilm-related infection diagnosis are based on (i) clinical wound characteristics: edema, erythema, warmth, and purulence; (ii) laboratory-based analyses: microbiological tests of wound swabs; and (iii) technical methods: scanning electron microscopy, gas chromatography–mass spectrometry, epifluorescence microscopy, colorimetric methods, and metabolic and biomass assays.^38^ In the context of AMR, adequate diagnosis and design of efficient treatments against biofilm-associated infection is critically needed.

### Topical antimicrobial and aseptic agents

**Figure 2.**
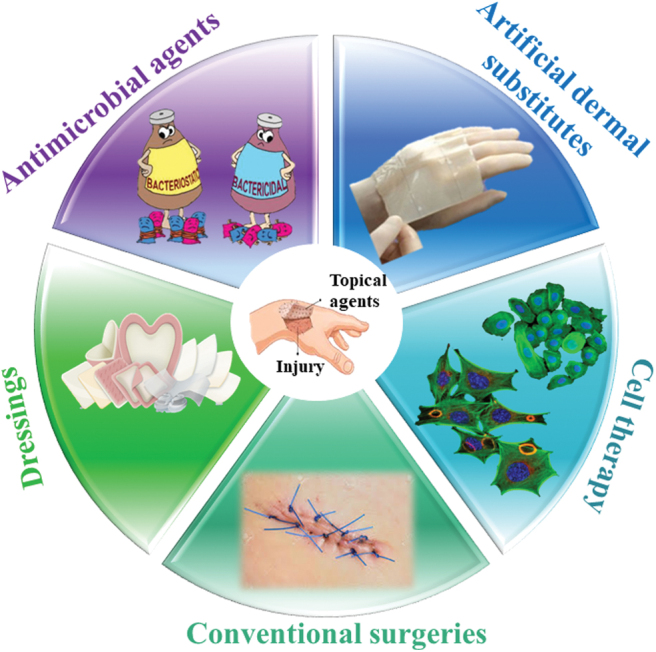
Current treatment methods for skin regeneration. Color images are available online.

Despite recent advances in wound management, very few topical therapies (Fig. 2) proved their efficacy in promoting wound healing. They led to a better understanding of factors influencing the process of wound healing and protection against bacterial infection. However, these methods have met with challenges, as well.^39,40^

Many wound dressings have been developed in an attempt to combine anti-infective properties and promotion of wound healing. Hydrophilic hydrogels and foams absorb wound exudates and keep the wound hydrated. However, they can act as a perfect breeding ground for bacterial growth. Bruises and scrapes result from frequent bandage change, which may lead to new skin injuries.^41^ Cotton gauze dressing has been considered for years as the standard of wound care, along with many other commercially available products, such as alginates, collagen dressings, regenerated cellulose, and honey, among others. However, the gold standard for treatment of chronic wounds is yet to be identified.^42^ Integration of antibiotics into conventional wound dressings has been of high interest.

Topical antimicrobial agents, including disinfectants, antiseptics, and antibiotics, have the ability to kill microorganisms, inhibit their growth, or reduce their number. Disinfectants are very potent against most microorganisms; however, their high toxicity toward all healthy tissues limits their application to inanimate objects and materials, such as surgical instruments and surfaces. Antiseptics have a broad antimicrobial activity and may be used even for some open wounds, but toxicity was reported as well.^43^

In contrast, antibiotics have high bactericidal activity through a specific cell-targeting mechanism—still limited by the AMR, which is very common nowadays. Moreover, administration of some antibiotics is accompanied by several side effects, such as pain, rash, cytotoxicity, toxic effects to kidneys, liver, and other organs.^44^ Several studies reported toxic encephalopathy-induced nonconvulsive status epilepticus,^45^ seizures,^46^ chronic myeloid leukemia, and renal failure,^47^ in the case of cephalosporin use.

Chronic wounds are treated using different topical antibacterial or antiseptic formulations with or without antibiotics as listed in Table 2, depending on the severity of the wound. Besides topical agents, treatment with dermal substitutes has shown effective to heal specific wounds. However, these substitutes generally lack the antimicrobial properties required for a durable outcome.

**Table 2.** Commercially available topical antimicrobials and antiseptics, their bacterial activity and limitations

TAA	Formulation(s)	Activity Against Microorganism(s)	Limitations	Ref.
Acetic acid	Solution 0.5%	Gram (+), (−) bacteria	Limited activity against biofilms; *in vitro* toxicity	^48^
Amoxicillin	Gel, solution	Gram (+), (−) bacteria	Resistance found	^49^
Bacitracin	Ointment	Gram (+), less active in Gram (−); resistance found in *Enterobacter* spp. and *Pseudomonas* spp.	Allergic reactions; may lead to overgrowth of drug-resistant organisms; cross-sensitization with neomycin; anaphylaxis	^50^
Cadexomer iodine	Gels, ointment, dressings	Gram (+), (−) bacteria; viruses; high wound exudates absorption	Cytotoxic; postapplication pain; renal failure; thyroid dysfunction; MRSA	^51,52^
Cephalosporins	Cream, gel, ointment	Gram (+), (−) bacteria	Renal dysfunction; gastrointestinal disorders; hematologic reactions; neurotoxicity; seizures; encephalopathy	^45–47^
Cerium nitrate	Cream, dressings	Gram (+), (−) bacteria	Methemoglobinemia; hematology alterations; weight loss	^53^
Chlorhexidine	Solution, sponge, brush, foam	Gram (+), (−) bacteria; less effective against *P. aeruginosa*	Cytotoxicity; allergic reactions, including anaphylaxis; resistance reported; injury to eyes and middle ear	^28^
Clotrimazole	Cream, ointment	Yeast; fungi	Recurring infection	^54^
Fusidic acid	Cream	Gram (+)	Rapid resistance; multiple applications per day	^55^
Gauze	Vaseline gauze, silicone gauze, sterile gauze	Nonbacterial	Wound drying; creates new injuries on changing; significant pain	^56^
Gentamicin	Cream and ointment	Gram (+), (−) bacteria	May lead to resistance; multiple application per day; ototoxicity	^57,58^
Honey	Dressings	Inhibits >50 bacterial species, including some MRSA	Nonmedical honey to be avoided (as it may contain spores)	^28,59,60^
H_2_O_2_	Solution, cream	Gram (+), (−) bacteria; fungi; viruses	Cytotoxicity; oxygen gas embolism	^61^
Mafenide acetate (Sulfamylon)	Cream, solution, ointment	Gram (+), (−) bacteria	Prone to cause pain upon application; allergic reactions	^62^
Mupirocin	Ointment	MRSA infection	Potential for developing resistance	^48^
Neomycin	Ointment, cream, powder	Gram (−) and some Gram (+) bacteria	Allergic contact dermatitis; may cause systemic toxicity; ototoxicity; nephrotoxicity	^63,64^
Neosporin	Ointment	Gram (+), (−) bacteria	Allergen	^65,66^
Nystatin	Cream	Fungi	Resistance reported	^65,67^
PHMB	Gel, solution, and dressing	Gram (+), (−) bacteria and fungi	Cytotoxicity; anaphylaxis	^68^
Polymyxin B (Colistin)	Ointment	Gram (−)	Last-resort; hypersensitivity reactions; neurotoxicity; renal acute tubular necrosis	^48,69^
Polysporin	Ointment	Gram (+), (−) bacteria and fungi	Potential for allergy if neomycin crosssensitization	^65,66^
Povidone/iodine	Solution, ointment, surgical scrub, cream, hydrogel	Gram (+), (−) bacteria; viruses; fungi; and yeast	Contact dermatitis; metabolic acidosis; delayed wound healing	^70–72^
Silver dressings	Foams, nanoparticle gel	Gram (+), (−) bacteria and fungi, including MRSA and VRE	Possible silver staining of tissues; delayed epithelialization (debated)	^28,42^
AgNO_3_	Cream, solution, sticks	Gram (+), (−) bacteria and fungi	Frequent reapplication due to short acting; methemoglobinemia; allergies; bacterial resistance	^48,73^
Silver sulfadiazine	Cream	Gram (+), (−) bacteria	Mild skin sensitiveness	^74,75^
Sodium hypochlorite	Dakin's solution	More active on Gram (+) than Gram (−) bacteria, fungi, and viruses	Cytotoxicity; postgraft bleeding; dissolve clots	^76,77^
Retapamulin	Ointment	Some Gram (+) and very few Gram (−) bacteria	May cause local reactions; several applications	^78^
Xeroform petrolatum	Dressing	Gram (+), (−) bacteria; yeast	Disputed antibactericidal activity	^79,80^
Zinc oxide-Scarlet Red	Fine mesh gauze, cream, ointment	Some Gram (+), (−) bacteria; fungi	Potential irritation	^48^

+, positive; −, negative; AgNO_3_, silver nitrate; H_2_O_2_, hydrogen peroxide; MRSA, methicillin-resistant *S. aureus*; PHMB, polyhexa-methylene biguanide; TAA, topical antimicrobials and antiseptics; VRE, vancomycin-resistant enterococci.

### Alternative agents to antibiotics

Nowadays, we face an urgent need to identify new antibacterial drugs to overcome AMR of different microorganisms.

#### Antimicrobial peptides

The AMPs, also referred as host defense peptides, were identified as good candidates to limit resistance-induced microorganisms. They are abundant in prokaryotes (produced by Gram-positive and Gram-negative bacteria) and in all eukaryotic organisms (fungi, algae, plants, insects, and mammals) and well distributed in cells and tissues as the front fighting line against pathogens.^81^ The first AMP, a tyrothricin compound was extracted from *Bacillus* strain by Dubos and Gause in 1939 independently from each other in their respective laboratories. It proved to be effective against pneumococci infection in mice.^82^ Soon after, it was found that the first AMP contained two different molecules: 80% tyrocidine and 20% gramicidin. Tyrocidine was very effective against both Gram-positive and Gram-negative bacteria, despite being highly toxic against mammalian cells.^83^ Gramicidin was applied for the treatment of infected wounds and especially ulcers during the Second World War.

Inspired by natural AMPs, many synthetic or semisynthetic analogs were recently developed. The main focus is on synthetic AMPs with higher antimicrobial activity and lower risks of toxicity toward host cells than their natural analogs. An online antibacterial peptide database (APD3) lists more than 3,130 AMPs originating from all species,^84,85^ out of which 134 are identified as human host defense peptides with more than 100 of those exhibiting antibacterial activity.^86^ Their classification depends on the charge, length, sequence of amino acids, and their secondary structure as shown in Table 3. They may have either amphiphilic or cationic domains, for example, human AMPs have a net charge range from anionic (rare) to cationic (most often), which ranges from −3 to +20.^86^

**Table 3.** The four antimicrobial peptide families with their type of conformation and examples

Family Type	Type of Secondary Structure	Examples	Ref.
α-Helix	α-Helical conformation	Cryptdin-4, human intestinal α-defensin HD5 and HD6, LL-37, Magainin 1 and 2, Moricin	^87,88^
β-Sheet	At least two β-sheets and two to four disulfide bridges	hBD-1, hBD-2, hBD-3, hBD-4, Pg1, Tachyplesin I	^88^
Loop	Single bond (either disulfide, amide, or isopeptide)	Thanatin	^88^
Extended family	Neither α-helical nor β-sheets	Indolicidin, Indolicidin analog (CP10A), Tritrpticin	^89^

hBD, human β-defensin; Pg1, protegrin-1.

Considering the critical situation of ESKAPE pathogens, AMPs are used to date as an effective therapy. They have the advantage of fast acting, bactericidal, multifunctional (stimulate the immune system and inhibit bacterial growth), and anti-inflammatory and/or wound healing promotor. AMPs, such as human β-defensins (hBD-1, hBD-2, and hBD-3)^90^ and cathelicidin LL-37,^91^ originating from epithelial tissues are factors of the innate immune system. They protect skin from infections caused by several microorganisms, such as *K. pneumoniae*, MRSA, *P. aeruginosa, Escherichia coli,* and *Neisseria gonorrhoeae*.^92^

Moreover, LL-37 is reported as a safe agent for clinical use as it successfully showed promotion of wound healing in hard-to-heal venous leg ulcers during short-term treatment.^93^ Histatin 5, human salivary peptide, has a strong antibacterial activity (≥70%) against five out of six ESKAPE pathogens, except *K. pneumoniae*. It also showed a strong *in vitro* antibiofilm formation in *P. aeruginosa* (60% killing) but less in *A. baumannii* and *S. aureus*.^94^

Cellular distress is usually noticed when exposing bacteria to antibiotics, although no bacterial adaptation nor resistance development was shown when treating *E. coli* for several hours with AMPs, such as cecropin A, melittin, magainin II, pexiganan, and LL-37 at 50% minimal inhibitory concentration (MIC). Also, treatment with these AMPs did not show any changes in mutation rate nor differential expression of genes related to stress-induced mutagenesis, while ampicillin, ciprofloxacin, and kanamycin antibiotics increased the mutation rate by threefold to fourfold.^95^

Bacterial resistance to AMPs and virulence was noticed in case of AMP proteolytic degradation. For instance, *S. aureus* together with an aureolysin metalloprotease could degrade LL-37 AMP by cleaving C-terminus bonds of the peptide and in turn contributing to resistance.^95^ ZapA metalloprotease could inhibit bacterial activity of LL-37 and hBD-1 against *Proteus mirabilis*, responsible for urinary tract infections, by at least 7- and 30-fold, respectively. Surprisingly, the same protease did not inhibit the activity of hBD-2, which has differences in amino acid sequence than hBD-1.^96^ *P. aeruginosa*, *Enterococcus faecalis*, and *Streptococcus pyogenes* use a common mechanism to escape α-defensins by secreting extracellular proteoglycans, which releases dermatan sulfate. Released compound further binds to α-defensins by completely inhibiting its activity.^95^

The process of biofilm formation is another mechanism of AMR to escape AMPs. It was reported that the DNA found in *P. aeruginosa* biofilm induces resistances to both polymyxin B and colistin by inducing lipopolysaccharide (LPS) modification.^95^ On the contrary, no resistance was found when treating protease-resistant *P. aeruginosa* biofilms with LL-37.^97^ In spite of these few reported resistance to pathogens, AMPs remain a promising tool to fight resistance, benefiting from their broad activity spectrum and the variety of their mechanisms of action.

### Antimicrobial peptides mechanism of action

Bacteria fall into two main categories depending on their cell wall structure: Gram-negative and Gram-positive bacteria. Gram-positive cells have an outer bacterial cell wall; whereas Gram-negative possess an additional outer membrane adapted with several porins and LPSs. These differences in the cell membrane will confer different susceptibility to various antimicrobial agents.^98^ For instance, some of the AMPs were reported to be more effective against Gram-negative than Gram-positive species.

Even though the mechanisms of action are currently debated among scientists, most agree on the key role of electrostatic forces between positively charged AMPs and negatively charged bacterial membrane, which in turns leads to bacterial leakage and death.^99^ Other proposed mechanisms are: (i) disruptive, such as “barrel-stave” and “toroidal pore” models of pore formation in the bacterial membrane; “carpet–detergent,” by which peptides can form micelles with the membrane components, (ii) nondisruptive, for example, bacterial membrane thinning, depolarization, or aggregation, and (iii) mediated by the “stringent response,” which is the stress response by the bacteria, involving secondary messenger metabolites.^98^ A detailed description of the modes of action is given by Bahar and Ren^100^ In addition, the AMP mechanisms of action depend on their concentration, *p*H, or temperature.^99,101^

### Antimicrobial peptides toxicity and efficacy: preclinical and clinical data

Despite their high and broad antimicrobial activity, AMPs may suffer from their toxicity toward mammalian cells. Toxicity against red blood cells (RBCs), or the ability of AMPs to lyse RBCs, also referred as hemolysis, is another major concern. Therefore, the selectivity toward bacterial cells is generally defined by the ratio of HC_50_/MIC, where HC_50_ is the concentration necessary to lyse 50% of RBCs and MIC is the minimal concentration to inhibit the growth of a given microorganism, for example, to obtain a bacteriostatic effect.^102^ Another important parameter is the minimal bactericidal concentration (MBC) of AMPs, which indicates the ability to eliminate (kill) bacteria. Antibacterial agents are usually regarded as bactericidal if the MBC is no more than four times the MIC.^103^

For the clinical use of AMPs, one should consider their mechanisms of action, stability under physiological conditions, and the balance between their efficacy and toxicity. Currently, only some AMPs with the ability to combat MDR bacteria have been approved by the FDA and already routinely used, such as Gramicidin (date of approval: 2005),^104^ Micafungin (2005),^105^ Anidulafungin (2006),^106^ Telavancin (2009),^106^ Ceftaroline (2010),^5^ Dalbavancin (2014),^106^ Oritavancin (2014),^106^ Caspofungin (2017),^106^ Ozenoxacin (2017),^5^ Tedizolid Phosphate (2015),^5^ and Omadacycline (2018).^5^ Most of them are against bacterial infection and administered either I.V. or topically.^107,108^ Some AMPs, not yet FDA approved, are being tested in clinical trials and listed in Table 4.

**Table 4.** Novel antimicrobial peptides for topical application tested in preclinical and clinical trials to eradicate multidrug-resistant bacteria

Peptide	Producer	Description	Application	Adminis-tration	Phase	Comments	Ref.
Brilacidin^®^	Innovation Pharmaceuticals, Inc.	Defensin mimetic	Acute skin and soft tissue infections in oral mucositis	I.V.	>II	Reduces oral mucositis in HNC patients	^109,110^
Dusquetide (SGX942)	Soligenix	First-in-class innate defense regulators	Oral mucositis	I.V.	III	Significantly reduces oral mucositis in HNC patients	^111^
hLF1–11	AM-Pharma	Lactoferricin-based peptide	Bacterial and fungal infections and for prophylaxis in hematopoietic stem cell transplantation	I.V.	I/II	Low antimicrobial efficacy and stem cell transplantation-associated infections in immunocompromised patients was reported; Company suspended trials	^112,113^
Human LL-37 (OP145)	ProMore Pharma	Human cathelicidin	Leg ulcer	Topical gel	>II	Significantly better than placebo	^93,114^
Lytixar (LTX-109)	Lytix Biopharma AM	Synthetic peptidomimetic	Skin infection; nasal colonization with *S. aureus* and impetigo	Topical hydrogel	I/II	Studies in progress	^115^
Novarifyn^®^ (NP432)	NovaBiotics	Synthetic peptide	MRSA, *P. aeruginosa, Clostridium difficile, A. baumannii, Escherichia coli*	Topical	PC	Ongoing studies	^116^
Novexatin	NovaBiotics	Cyclic cationic peptide	Fungal nail infection	Topical brush	II	Ongoing studies. No side effects reported yet	^114^
PAC-113	Pacgen; Demegen	Synthetic histatin 3	Oral mouth rinse for Candidiasis in HIV patients	Topical solution	II	High efficacy for oral candidiasis	^117^
Pexiganan (Locilex^®^, MSI-78)	Magainin Pharmaceuticals	Magainin 2 analog	Diabetic foot ulcer	Topical cream	III	One of the debated peptide for its poor antibacterial efficacy compared with other antibiotics	^16,26,118,119^
Polymyxin	Athenex	Cyclic cationic lipopeptides	Urinary tract infection, mucositis, ocular and wound infection treatment	I.M., I.T., I.V., ophthalmic	II/III	Used as a “last resort” due to its high toxicity (neurotoxicity and nephrotoxicity); excluded from CLSI list	^120,121^

hLF, human lactoferrin fragment; HNC, head and neck cancer; I.M., intramuscular; I.T., intrathecal; I.V., intravenous injection; PC, preclinical.

Most of the AMPs cannot reach the clinical phase due to their systemic toxicity, fast degradation, short half-life, and/or reduced activity in the presence of salts or divalent cations.^122^ Several AMPs in clinical trials failed to show a better activity than conventional antibiotics or exhibited adverse side effects, although in general one should consider the trade-off between toxicity and efficacy. Some examples are Iseganan (withdrawn after phase III) intended for the treatment of oral mucositis^117,123^ and Omiganan (withdrawn after phase III), a topical gel for prevention of catheter infections, acne and rosacea.^122,124^ These failures have spurred the development of encapsulation strategies of AMPs into different delivery systems, such as nanoparticles and liposomes, to enhance their stability and half-life.

Besides clinically approved antimicrobial and antiseptic agents, synthetic dendritic polymers and peptide dendrimers have recently shown promising developments, detailed in the dedicated paragraph below. In addition, other approaches such as encapsulation into nanocarriers or chemical coupling to other molecules are used to reduce AMPs toxicity and increase their efficacy.

#### Polymer dendrimers for topical application

##### Poly(amidoamine)

**Figure 3.**
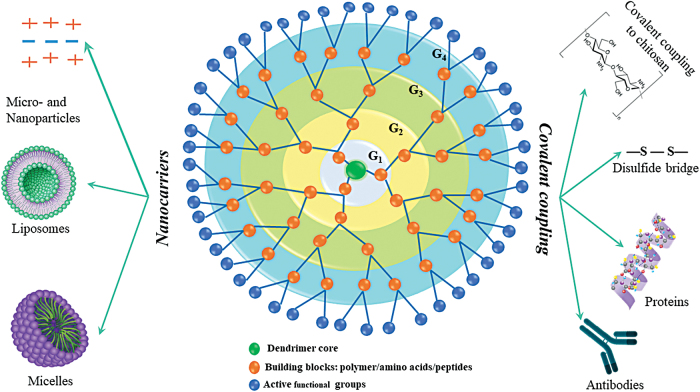
Schematic representation of branched dendrimers and their delivery systems. Color images are available online.

Synthetic poly(amidoamine) (PAMAM) dendrimers are available up to the 10th generation and mostly studied for their possible antimicrobial efficacy. The most common polymeric dendrimers are PAMAM, polypropyleneimine, poly-l-lysine, carbosilane, polyglycerol, poly(bencyl ether), and phosphorus dendrimers.^125^ Polymer dendrimers have a three-dimensional structure with different density of functional groups and have been found as effective antibacterials. Starting from the inner core of the dendrimer toward the external side, each step of ramification is identified as a generation (G_x_), as illustrated in Fig. 3.

The modification of one unit will affect the properties of the whole dendrimer construct. Calabretta *et al.* have reported for the first time the effectiveness of fifth-generation (G_5_) amino-terminated PAMAM dendrimers against both *P. aeruginosa* and *S. aureus* at very low concentrations (MIC of 1.5 and 20.8 μg/mL, respectively).^126^ However, G_5_ PAMAM exerted higher toxicity (25% survival at 10 μg/mL) toward human corneal epithelial cells compared with LL-37 (significant toxicity at 25 μg/mL), potentially due to the highly branched cationic dendrimers.

Interestingly, a smaller PAMAM generation (G_3_) was found to have an enhanced activity against *P. aeruginosa* and *S. aureus* when compared with G_5_ PAMAM (G_3_: 6.3 μg/mL vs. G_5_: 12.5 μg/mL), or LL-37 (1.3–12.5 μg/mL).^127^ This suggests that the number of amino groups displayed by the dendrimers—higher for G_5_ than for G_3_—is not the sole factor for the antimicrobial activity observed.

PEGylation of the functional groups in PAMAM dendrimers was reported to reduce both the toxicity and bactericidal activity, while complete polyethylene glycol replacement of functional groups inhibited the activity of the dendrimer against *P. aeruginosa*, most probably due to decreased number of amino groups.^126,127^ Another approach to reduce the toxicity of PAMAM dendrimers is to modify the functional groups into amino-, hydroxyl-, and carboxyl-terminated G_4_-PAMAM.^128^

The antibacterial activity against *E. coli in vitro* was found to decrease from G_4_-PAMAM-NH_2_ to G_4_-PAMAM-OH to G_4_-PAMAM-COOH (IC_50_ of 3.8 μg/mL, 5.4 mg/mL, and 22 mg/mL, respectively). Topical vaginal and cervical application of G_4_-PAMAM-OH in a pregnant guinea pig model of chorioamnionitis (intrauterine infection by *E. coli*) lead to major changes to the outer membrane of *E. coli*, while G_4_-PAMAM-NH_2_ induced changes to both inner and outer bacterial membranes.^128^ However, G_4_-PAMAM-NH_2_ was dropped for further potential application due to its very high toxicity, while G_4_-PAMAM-OH was barely transported across placental membrane model, suggesting safety for pregnant women.^129^

Lower generation, G_1_ PAMAM-disaccharide galabiose modified exhibited a 3,000-fold increased potency against *Streptococcus suis* with an MIC of 0.3 nM. G_1_ dendrimer was able to inhibit the adhesion of *S. suis*.^130^ Actually, increasing the number of generations in amino-PAMAM from G_3_ to G_7_ significantly decreased *in vitro* viability and inhibited differentiation of human neural progenitor cells and damaged DNA at a concentration of 5 μg/mL. In contrast, G_0_, G_1,_ and G_2_ at the same concentration did not show any cytotoxicity.^131^ Therefore, lower generation PAMAM hold promise to improve the efficacy–toxicity ratio of the dendrimers, paving the way to clinical applications.

##### AMPDs for topical applications

**Figure 4.**
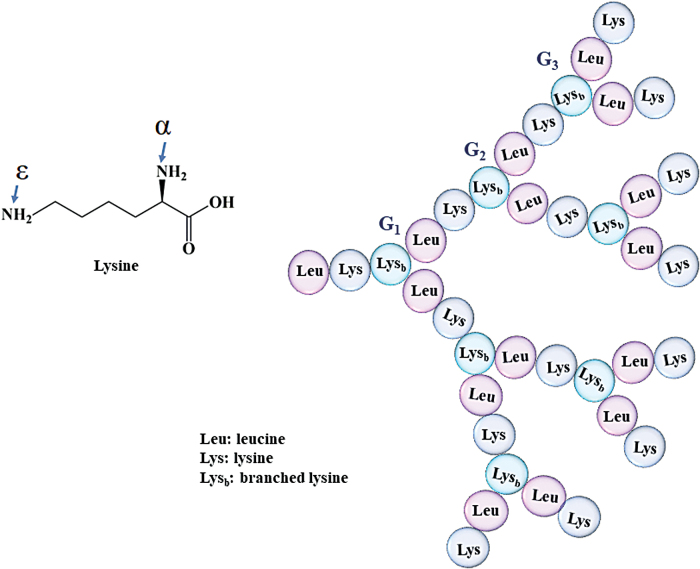
Amino acid sequence in AMPD (*e.g.*, G3KL), which is based on a divalent lysine core whose α and ɛ amines along with leucine double geometrically with each ramification building up a new generation. AMPD, antimicrobial peptide dendrimer. Color images are available online.

Compared with linear AMPs, AMPDs show a three-dimensional, regularly branched structure built by covalent bonds, a very low polydispersity and a higher density of surface groups.^17^ Their structure is very similar to the polymeric dendrimers, except that they have only one-side branches, which makes them more flexible for chemical coupling or incorporation into different delivery systems (Fig. 4). The synthetic flexibility and high density of the functional groups found in AMPDs make them very attractive for use as delivery systems for drugs and bioactive principles. Designing peptide-based agents is strongly supported by the high potency of the AMPDs not only to kill bacteria, but also to reduce the toxicity against mammalian cells.^132^

The displayed functional groups will govern the mechanism of bacterial killing. AMPDs, which bear charged ends, are believed to act by penetrating the cell membrane inducing leakage of intracellular materials resulting in bacterial death. Therefore, the AMPD mechanism of action against bacteria is related to the number of functional surface groups and their ability to cross the cell membrane.^132^

AMPDs show increased activity, which is usually attributed to the higher local concentration of bioactive units in such assemblies, and to their greater stability against peptidases and proteases. For instance, dendrimeric peptides were shown to be selective for microbial surfaces with a broad antimicrobial and low hemolytic activity. A family of AMPDs based on R4 tetrapeptide (RLYR) and R8 octapeptide (RLYR-KVYG), were tested against 10 different microbial strains. Both R4 and R8-based dendrimers of fifth and eighth generation exhibited high activity with MICs <1 μM against Gram-positive and Gram-negative bacteria as well as fungi.^133^

Besides, these AMPDs were resistant to proteolytic degradation or to protease inhibition, which has been attributed to their dendrimeric structure. A lipodimeric peptide, SB056, was investigated for its antimicrobial activity against a range of bacteria, including Gram-positive and Gram-negative strains. The *in vitro* assays showed high antimicrobial activity with MIC in the range of 2–32 μg/mL against *A. baumannii*, *Enterobacter cloacae*, *E. coli*, *K. pneumoniae*, and *P. aeruginosa*, which is comparable with the activity of polymyxin B.

The SB056 AMPD showed strong activity against *E. coli* and *S. aureus* strains as well as strong *Staphylococcus epidermidis* biofilm inhibition.^134^ Further improvements on the amphipathic part of the SB056 resulted in more ordered β-strands with a stronger antimicrobial activity against both Gram-positive and Gram-negative bacteria.^135^ A study on series of tryptophan-ending dendrimers showed that amphiphilic AMPDs can be an effective therapy of *E. coli* infections. Most of the tryptophan-anchored AMPDs were able to inhibit the growth of antibiotic-resistant *E. coli* strains, sometimes better than polymyxin B or even indolicidin, besides showing stability in plasma along with low hemolysis and genotoxicity.^136^

Recently, a novel G3KL (containing repetitive units of lysine and leucine) AMPD showed high potency at low MIC against 35 strains of *P. aeruginosa* (8–32 μg/mL), 32 strains of *A. baumannii* (16 μg/mL), *E. coli* (8 μg/mL), and *K. pneumoniae* (16–64 μg/mL).^17^ G3KL is a peptide dendrimer of third generation, which acts as a membrane-disrupting agent against bacteria. G3RL (with repetitive units of arginine and leucine) showed lower efficiency than G3KL against *P. aeruginosa* (8–32 μg/mL) and *Bacillus subtilis* (11 μg/mL). Once in contact with the serum, the biological activity of G3RL is inhibited.^132^

Moreover, both G3KL and G3RL within biological bandages have shown high efficacy against *P. aeruginosa*, absence of toxicity, and no gene alteration in progenitor fibroblast cells at a concentration of 100 μg/mL. Especially G3KL showed enhanced angiogenesis in human umbilical vein endothelial cells and chorioallantoic membrane assays, as a proof of further potency to enhance wound healing.^137^ A second-generation AMPD, such as TNS18, has the same activity as G3KL against Gram-negative bacteria, except *K. pneumoniae* and against Gram-positive MRSA (MIC = 8–16 μg/mL).^17^

Moreover, D-enantiomeric dendrimers dG3KL and dTNS18 have shown high killing effect against different *P. aeruginosa* biofilm strains (90.2–100%) *in vivo* on larvae.^138^ Therefore, the topology and the sequence of the dendrimers can not only affect their antimicrobial potential, but can also alter their proangiogenic effect, as well. Moreover, the same group of Reymond have developed two different glycopeptide dendrimers: a fucosylated peptide dendrimer (FD2) and two galactosylated dendrimer (GalAG2 and GalBG2), which proved to be potent against *P. aeruginosa* biofilm formation *in vitro*.^139^

The AMPDs show strong potency against multiple bacterial strains and biofilms. Further research is warranted to optimize their delivery to the wounded site, for a potential clinical translation.

### Delivery strategies for AMPs

The limitations of AMPs in terms of efficacy, fast degradation, or toxicity require adequate delivery strategies to tackle these challenges. As AMPDs are a relatively new class of antimicrobials, most of the research has been performed on AMPs, which were covalently coupled to biopolymers or encapsulated into nanoparticles or liposomes. The design of AMP nanocarriers could serve as an example of how to render AMPDs even more effective while preserving their bioactivity.

### Covalent coupling of AMPs to chitosan–chitosan derivatives

AMPs can be favorable drug candidates to be coupled to biopolymers, such as chitosan or chitosan derivatives to reduce hemolytic effects and/or enhance antibacterial activity, also benefiting from chitosan's bacteriostatic properties. These conjugates have the advantage of increased stability against proteases and peptidases, low immunogenicity, high efficiency and selectivity, and relatively small size that allows AMPs to disrupt bacterial wall.^140^

- Anoplin–chitosan: Anoplin (derived from wasp venom) covalently coupled to chitosan showed enhanced *in vitro* bioactivity and absence of hemolysis. The activity of anoplin–chitosan conjugates against *S. aureus* and *E. coli* increased proportionally with their degree of substitution (MIC of anoplin peptide of 1.9 μg/mL against *E. coli*).^141^
- HHC10–chitosan: Cysteine-HHC10 AMP coupled to chitosan showed enhanced bioactivity against *S. aureus* and *S. epidermidis*; almost no hemolysis and lower toxicity than HHC10 alone.^142^
- hLF1–11–chitosan: Human hLF1–11 covalently coupled to a thiol-derivatized chitosan film lead to a significant increase in *S. aureus* adhesion against implant-related infections.^143^
- Dhvar-5–chitosan: This peptide was immobilized to chitosan films for *S. aureus* elimination.^144^

These studies suggest a potential for chitosan–peptide conjugates to improve activity and decrease toxicity compared with the parent peptide. Still, we need further investigations to validate this experimental approach and reveal the mechanisms behind this improvement.

### Nanocarrier systems

Encapsulation of peptides into nano- or microcarriers systems can be an efficient approach to lower cytotoxicity, preserve activity by reducing their degradation and enhance their selectivity.^145^ Among these, liposomes, micelles, polymer nanoparticles, and microparticles have met success for drug delivery. This approach has been applied to some AMPs, potentially improving their pharmacokinetic profile.

#### Liposomes

Liposomes are self-assembled colloidal systems composed of one or more phospholipid bilayers. They have been studied in the last decades as suitable vehicles for drug delivery due to their encapsulation ability and biocompatibility.^145^ Liposomes have the advantage of encapsulating both hydrophobic and hydrophilic compounds. Moreover, these drug release systems may protect AMPs against degradation, decrease cytotoxicity, and enhance their stability and bioactivity. For example, Yang *et al.* incorporated a WLBU2 peptide (24-amino acids) using a modified liposome delivery system with high efficiency against both Gram-positive and Gram-negative bacteria (*P. aeruginosa* and *S. aureus*, respectively) and against *Chlamydia trachomatis*.

Furthermore, WLBU2-modified liposomes were safe to human skin fibroblasts and the activity of the peptide was preserved even in the presence of human serum and blood.^146^ This AMP-modified liposome system could be potentially used for local infections. An I.V. injection of tuftsin-loaded liposomes in infected animals resulted in site-specific delivery of AMP and stimulation of liver and spleen macrophage functions against antibacterial–antiparasitic infections, such as tuberculosis and leishmaniasis.^147^

Vancomycin and chitosan-loaded liposomes were shown to not only improve the pharmacokinetic profile of the peptide, but to also reduce nephrotoxicity in mice. Injectable vancomycin liposomes showed high antibacterial efficacy against Gram-positive bacterial infections, a sustained release profile, and prolonged systemic circulation.^145^ This prevented a vancomycin burst release, which could lead to different side effects.

#### Micelles

A DP7 (12-amino acids) cationic and hydrophilic AMP, incorporated into a micellar system, showed potent therapeutic benefits in different *in vivo* disease models and proved to be safe through I.V. injection in mice. The AMP–micellar construct showed reduced hemolysis and high antibacterial activity against *S. aureus* both *in vitro* (MIC of DP7-micelles >1,024 μg/mL against *P. aeruginosa*, *S. aureus*, and *E. coli*) and *in vivo*.

In *P. aeruginosa*-infected zebrafish embryos and *S. aureus*-infected mice, DP7-micelles showed high efficacy and therapeutic safety comparable to vancomycin. After an I.V. (80 mg/kg body weight) administration of DP7-micelles, all mice survived and no liver bleeding or pulmonary hemorrhage was observed.^148^ These AMP-micelle formulations may potentially be used for bacterial infections (in both Gram-positive and Gram-negative species) as they were shown to significantly stimulate defensive immune reactions *in vivo* as well.

#### Micro- and nanoparticles

Vancomycin has been loaded into polycaprolactone polymer microparticles to minimize the side effects of vancomycin. These microparticles were coadministered with calcium phosphate bone substitutes for preventing postsurgery infection. The encapsulation of vancomycin into microparticles resulted in prolonged peptide release *in vitro* over several weeks.^149^ Vancomycin was successfully encapsulated into poly(lactide-*co*-glycolide) (PLGA) polymers to prevent external–internal ocular bacterial infections.^150^ Thus, vancomycin encapsulated into nanodelivery systems may successfully be used as an alternative treatment of infections caused by MDR bacteria.

Piras *et al.* could formulate an efficient nanoparticles system against *S. epidermidis* by ionic gelation method. Peptide LLPIVGNLLKSLL-amide (called TB) was added to chitosan to form NPs. The encapsulation of TB peptide exhibited high bactericidal properties against *S. epidermidis* strains and significantly reduced the toxicity against mammalian cells.^151^ Another RBRBR peptide was encapsulated into chitosan NPs by a similar method resulting in an enhanced activity against *S. aureus* and significantly reduced hemolysis and cytotoxicity.^152^ Thus, chitosan may act as an antimicrobial activity enhancer and/or significantly limit the toxicity of the AMPs.

d'Angelo *et al.* engineered a colistin-loaded PLGA nanoparticles system for sustained delivery of the peptide against *P. aeruginosa* in lung infection. Colistin encapsulated into PLGA NPs could easily penetrate an artificial mucus layer during the first 6 h and successfully eliminated *P. aeruginosa* biofilm *in vitro* within 72 h at 7.5 and 15 μg/mL.^153^

These few studies of AMPs covalently coupled or noncovalently associated to different biopolymers suggest that AMPs are potent candidates to eradicate MDR bacterial infections at an enhanced antimicrobial activity and lower toxicity. Additionally, achieving optimum drug–AMP loading, using the right and safe polymer, storing the new formulation while preserving the bioactivity and stability of the AMP are definitely to be further evaluated.

## Summary

The occurrence of AMR has changed the landscape of the drugs used in clinics, more specifically to treat ESKAPE-related infections. In addition, the complexity of the factors affecting wound healing renders the choice of adequate antimicrobial agents difficult. In this context, alternative strategies to overcome AMR are proposed.

Besides clinically approved antimicrobial and antiseptic agents, synthetic dendritic polymers and novel peptide dendrimers (AMPDs) have recently shown promising results in preclinical models of infection. Further strategies are also available to improve their activity and decrease toxicity compared with the parent peptides: the conjugation with (bio)polymers, or the incorporation into carriers such as liposomes, nano- or microparticles. These strategies may allow for a sustained pharmacokinetic profile and improve the activity against MDR bacterial infections, paving the way toward their use in a clinical setup.

**Take-Home Messages**

- The occurrence of AMR has changed the landscape of the drugs used in clinics, more specifically to treat ESKAPE-related infections.
- Antimicrobial peptidic agents are highly potent with a broad activity against Gram (+) and (−) bacteria and microorganisms.
- AMPs are good candidates to limit resistance-induced microorganisms, benefiting from their broad activity spectrum and the variety of their mechanisms of action.
- The performance of AMPs can be further enhanced by several strategies such as: chemical conjugation to biopolymers or organization in a dendritic structure.
- Nanocarrier technology may further improve pharmacokinetic profile to increase antimicrobial effect and reduce toxicity.
- AMPDs show strong potency against multiple bacterial strains and biofilms. Further research is warranted to optimize their delivery to the wounded site, for a potential clinical translation.

## About the Authors

**Yayoi Kawano, PhD,** is Associate Professor at Faculty of Pharmaceutical Sciences, Tokyo University of Science, Japan. Her research interests are to incorporate poorly water-soluble drugs into different drug delivery systems, and to evaluate formulations for wound healing. **Olivier Jordan, PhD,** is Senior Lecturer at the University of Geneva. His research interests lie in the field of innovative carriers for drug, peptide, or protein delivery based on biopolymers, including hyaluronic acid or chitosan. **Takehisa Hanawa, PhD,** is Professor at the Faculty of Pharmaceutical Sciences of Tokyo University of Science. His research interests are to prepare and apply nanoparticle, hydrogel, and film formulations for various external formulations. **Gerrit Borchard, PharmD, PhD,** is a Full Professor in Biopharmaceutics at the University of Geneva. His group s research focuses on the interaction of drug and vaccine formulations with biological systems, including immunotherapy of cancer and infectious diseases, development and characterization of nanomedicines applied in these indications. **Viorica Patrulea, PhD,** is a postdoctoral fellow at the University of Geneva. She has developed a new technological platform based on her expertise in polymer chemistry and complemented by the knowledge of biological and pharmaceutical aspects of the development of formulations for wound healing and protection against microbial infection.

## Abbreviations and Acronyms

AgNO_3_: silver nitrate
AMP: antimicrobial peptide
AMPD: antimicrobial peptide dendrimer
AMR: antimicrobial resistance
CLSI: Clinical and Laboratory Standards Institute
ESKAPE: *Enterococcus faecium, Staphylococcus aureus, Klebsiella pneumoniae*, *Acinetobacter baumannii*, *Pseudomonas aeruginosa*, and *Enterobacter* species
FDA: U.S. Food and Drug Administration
Gram (+): Gram-positive
Gram (−): Gram-negative
H_2_O_2_: hydrogen peroxide
hBD: human β-defensin
HC: hemolytic concentration
hLF: human lactoferrin fragment
HNC: head and neck cancer
I.M.: intramuscular
I.T.: intrathecal
I.V.: intravenous injection
LPS: lipopolysaccharide
MBC: minimal bactericidal concentration
MDR: multidrug resistant
MIC: minimal inhibitory concentration
MRSA: methicillin-resistant *S. aureus*
PAMAM: poly(amidoamine)
PC: preclinical
Pg1: protegrin-1
PHMB: polyhexa-methylene biguanide
PLGA: poly(lactide-*co*-glycolide)
RBC: red blood cell
TAA: topical antimicrobials and antiseptics
VRE: vancomycin-resistant enterococci
WHO: World Health Organization
